# The Effect of Neurobehavioral Test Performance on the All-Cause Mortality among US Population

**DOI:** 10.1155/2016/5927289

**Published:** 2016-08-10

**Authors:** Tao-Chun Peng, Wei-Liang Chen, Li-Wei Wu, Ying-Jen Chen, Fang-Yih Liaw, Gia-Chi Wang, Chung-Ching Wang, Ya-Hui Yang

**Affiliations:** ^1^Division of Family Medicine, Department of Family and Community Medicine, Tri-Service General Hospital and School of Medicine, National Defense Medical Center, Taipei 114, Taiwan; ^2^Division of Geriatric Medicine, Department of Family and Community Medicine, Tri-Service General Hospital and School of Medicine, National Defense Medical Center, Taipei 114, Taiwan; ^3^Graduate Institute of Medical Sciences, National Defense Medical Center, Taipei 114, Taiwan; ^4^Department of Occupational Safety and Hygiene, Fooyin University, Kaohsiung 114, Taiwan; ^5^Department of Education and Research, Kaohsiung Veterans General Hospital, Kaohsiung 114, Taiwan

## Abstract

Evidence of the association between global cognitive function and mortality is much, but whether specific cognitive function is related to mortality is unclear. To address the paucity of knowledge on younger populations in the US, we analyzed the association between specific cognitive function and mortality in young and middle-aged adults. We analyzed data from 5,144 men and women between 20 and 59 years of age in the Third National Health and Nutrition Examination Survey (1988–94) with mortality follow-up evaluation through 2006. Cognitive function tests, including assessments of executive function/processing speed (symbol digit substitution) and learning recall/short-term memory (serial digit learning), were performed. All-cause mortality was the outcome of interest. After adjusting for multiple variables, total mortality was significantly higher in males with poorer executive function/processing speed (hazard ratio (HR) 2.02; 95% confidence interval 1.36 to 2.99) and poorer recall/short-term memory (HR 1.47; 95% confidence interval 1.02 to 2.12). After adjusting for multiple variables, the mortality risk did not significantly increase among the females in these two cognitive tests groups. In this sample of the US population, poorer executive function/processing speed and poorer learning recall/short-term memory were significantly associated with increased mortality rates, especially in males. This study highlights the notion that poorer specific cognitive function predicts all-cause mortality in young and middle-aged males.

## 1. Introduction

Low cognitive function, a state that involves problems with memory, learning, thinking, language, complex attention, and executive function, has been associated with cardiovascular and cerebrovascular disease and cancer [1–5]. Previous population-based studies have assessed the role of cognitive function as a predictor of all-cause mortality [6–9], cause-specific mortality [10], or both [11, 12] in elderly populations, and emerging evidence also demonstrates an increased risk of mortality among childhood and early adulthood patients with cognitive impairment [13–15]. Studies have examined young and middle-aged populations comparatively less. Elderly subjects generally have higher rates of comorbidities or subclinical illnesses, which may confound the risk of death compared to younger and healthier subjects. Therefore, these studies that have focused on elderly subjects are not generalizable to a broader population of younger subjects. In addition to age, differences exist in measurements (e.g., using either global cognitive function tests or different aspects of cognitive domain tests) in evaluating the association with mortality. To date, little work has been done on which predominant cognitive domain is associated with mortality. Reaction time is one of the predictors of mortality [16–19]. One study by Hagger-Johnson et al. [19] demonstrated that reaction time is related to mortality in the Third National Health and Nutrition Examination Survey (NHANES III) sample. Despite also the important neurobehavioral performance measurement, the other two components in NHANES III, that is, digit symbol substitution test (DSST) and serial digit learning task (SDLT), have been less focused on. Previous studies with relatively short follow-up periods and older population have shown that lower DSST scores are also a risk factor for mortality [7, 10, 20]. However, few studies have examined the link between specific cognitive functions and mortality risk among large-scale young and middle-aged US populations. To further investigate this potential relationship, we analyzed the results of NHANES III. Furthermore, we explored gender-specific effects because sex-related mortality difference had been noted in Alzheimer's disease [21] and cognitive impairment [22]. Nevertheless, a paucity of studies have focused on potential gender differences with regard to the association between cognitive function in various domains and mortality risk.

## 2. Materials and Methods

### 2.1. Study Population and Data Collection

We selected adults between 20 and 59 years of age who were enrolled in the NHANES III survey, a stratified multistage clustered probability survey conducted in a noninstitutionalized US population, and had data from at least one cognitive test available (*n* = 5,144). A detailed description of the NHANES III survey methodology has been published [23].

Two computerized cognitive tests from the Neurobehavioral Evaluation System (NES) [24] were collected. The symbol digit substitution test (SDST), also named digit symbol substitution test (DSST), measures executive function and processing speed. Subjects were instructed to recognize a grid on the upper half of a computer screen that displayed unique symbols matched to particular numbers (one to nine). Subjects were then asked to press the matched number for each symbol as soon as possible after a symbol was displayed on the computer screen. The latency time was recorded in seconds, and the number of correct responses was quantified. This summary measure was recorded as “sec/correct digit.” Higher values on the SDST indicate poorer performance. The SDLT measures learning recall and short-term memory. Subjects were required to remember a series of numbers and then recall the entire sequence of numbers in the correct order. The count score of serial digit learning total errors was recorded. Higher scores on the SDLT indicate poorer performance. Details regarding the methodology and scoring rules for the NES have been previously reported [24]. Each cognitive test score was divided by the standard deviation (SD) of the increase or decrease in the cognitive test scores. The subjects in the “1 SD increase” group were categorized as the “inferior” group.

Additional covariate data were collected. Age was calculated from the date of birth to the date of the interview. Race/ethnicity was grouped according to the following categories: non-Hispanic White, non-Hispanic Black, Mexican American, and other ethnicities. Gender was self-reported as either male or female. Body mass index (BMI) was calculated by dividing the individual's weight in kilograms by the square of their height in meters. Education was categorized as either less than high school (≤grade 9) or greater than or equal to high school. Blood pressure was measured using a mercury sphygmomanometer. Participants who answered “yes” to the question “Have you smoked 100+ cigarettes in your life?” were classified as smokers. Medical conditions (including self-reported congestive heart failure, chronic bronchitis, and stroke) were also recorded. C-reactive protein was measured using latex-enhanced nephelometry. Plasma glucose was measured using an enzymatic reaction assay. The serum folate concentration was measured using a commercial radioimmunoassay kit. The NHANES III study received approval from the National Center for Health Statistics Institutional Review Board; written informed consent was obtained from participants before starting the study.

### 2.2. Data Analysis

Our primary outcome of interest was all-cause mortality. We used the NHANES III (1988–1994) Linked Mortality Files from the National Center for Health Statistics, which provided mortality follow-up data from the date of the NHANES III survey participation through December 31, 2006. The NHANES III mortality linkage was used to link participants to mortality data in the National Death Index (NDI). Participants were censored at the end of the follow-up assessment or at death. Additional details information regarding the probabilistic matching technique employed by the National Center for Health Statistics has been published [25]. Stratified analysis by sex was conducted. Kaplan-Meier survival curves were plotted to ascertain the relationship between the different cognitive tests and subsequent mortality. Cox proportional hazards regression analyses were performed to calculate the hazard ratio (HR) of the association between cognitive function variables and all-cause mortality. Model 1 was the unadjusted model. Model 2 was adjusted for age, race/ethnicity, education, blood pressure, serum folate, serum C-reactive protein, serum glucose, BMI, smoking status, heart failure, stroke, and chronic bronchitis. A *P* value less than 0.05 was considered statistically significant. All statistical analyses were performed using SPSS (Version 18.0 for Windows, SPSS, Inc., Chicago, IL, USA).

## 3. Results

A total of 5,144 participants, with a mean age of 37 years, were included in the study. There were 380 subjects who died during the mean follow-up period of 15 years.  Table 1 shows the demographic and clinical characteristics of the survivor group, deceased group, and the entire population. Participants in the deceased group were more likely to be male, to be smokers, to have a low educational level, and to have a chronic disease compared to the survivor group. The mean blood pressure, serum glucose, C-reactive protein, and BMI were higher among the deceased group than the survivor group.

**Figure 1:**
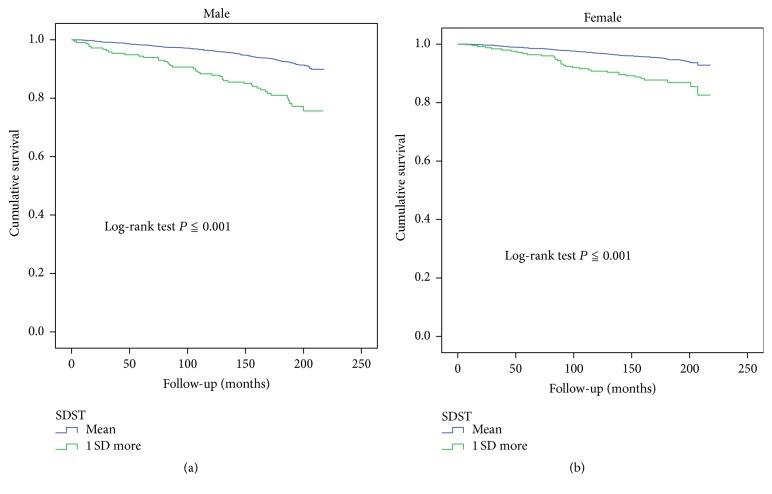
(a) Kaplan-Meier plot of the association between SDST groups and mortality in males. (b) Kaplan-Meier plot of the association between SDST groups and mortality in females.

**Figure 2:**
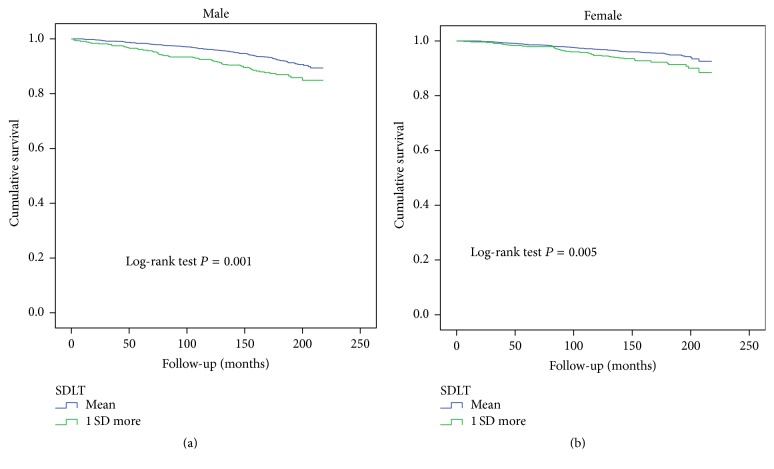
(a) Kaplan-Meier plot of the association between SDLT groups and mortality in males. (b) Kaplan-Meier plot of the association between SDLT groups and mortality in females.

Kaplan-Meier analyses stratified by the 2 test groups demonstrated a trend toward higher mortality among male participants with 1 SD increases in their SDST and SDLT scores (log-rank test: *P* < 0.001 for all comparisons) compared with those who had mean scores (Figures 1(a) and 2(a)). In female participants with 1 SD increases in their SDST and SDLT scores, a similar trend was observed (log-rank test: *P* < 0.001, *P* = 0.002, and *P* < 0.001, resp.; Figures 1(b) and 2(b)).

To further examine the association between cognitive function and mortality while controlling for potential confounders, the HR (95% confidence interval) for all-cause mortality was calculated for each cognition test and compared using the Cox proportional hazards model as shown in Table 2. In model 1 (unadjusted), male and female participants with 1 SD increases in their SDST and SDLT scores were significantly associated with increased mortality (all *P* < 0.05). In model 2 (multivariate-adjusted), which was adjusted for age, race/ethnicity, education, blood pressure, serum glucose, serum folate, C-reactive protein, smoking, and chronic disease, these ratios remained significant for male participants with 1 SD increases in their SDST and SDLT scores (*P* = 0.001 and 0.041, resp.); however, the HRs for female participants with 1 SD increases in their SDST and SDLT scores were not significant after multivariate adjustment. The main confounding factors in the association between exposure and outcome in model 2 of SDST in women are age, smoking, heart failure, and chronic bronchitis. The main confounding factors in the association between exposure and outcome in model 2 of SDLT in women are age, smoking, glucose, heart failure, and chronic bronchitis.

## 4. Discussion

This study of a large representative United States sample demonstrated that younger male subjects with poorer processing speed or short-term memory had a significantly higher risk of all-cause mortality. This association was still robust even after adjusting for education, chronic disease, and other latent confounders. However, in the female participants, none of the two cognitive measures were associated with increased mortality in the fully adjusted models.

Our study corroborated and extended prior research showing that poorer cognitive function increased the risk of mortality and that this risk was also recognizable in younger adults for whom dementia is considerably less common. Few studies have investigated the association between cognitive function and mortality among young and middle-aged US populations. In the Whitehall II cohort study of 10,308 civil servants aged 35–55 years in London, there was a substantially increased risk of mortality among middle-aged populations with impaired cognitive domains of short-term memory and reasoning [26]. In addition, a cohort study conducted by Shipley et al. [27] found that reaction time and memory ability were related to all-cause mortality for the entire age range, and Pavlik and colleagues [10] noted that poor performance on memory and attention tests was associated with increased mortality in middle-aged individuals. Schmidt et al. [3] studied young adults in Denmark and showed that low cognitive scores strongly predicted death. Our findings are in line with these previous research studies, showing that poorer processing speed or short-term memory is related to an increased mortality risk in a younger population, especially males. A recent study of the NHANES data also found that slower reaction times are associated with increased risk of mortality [19]. Using the same dataset, but applying two different cognitive tests and a gender-specific analysis, we found a slightly different conclusion.

An additional interesting finding of our study is that the presence of poorer executive function/processing speed/learning recall/short-term memory did not increase the risk of mortality among females. This study cannot explain the underlying causal mechanisms of the observed result. A variety of factors might contribute to this finding including sociocultural factors [28], the protective effect of estrogen, or the presence of fewer comorbid clinical conditions [29]. In a study of more than 23,000 US participants aged 60 years or older, women showed longer median survival times after an initial diagnosis of cognitive impairment [21]. In a cognition-mortality link study by Schultz-Larsen et al., a subscale of the MMSE was a poor predictor of mortality in women [22]. These previous studies were conducted in older patients diagnosed with dementia. Few studies have focused on the gender differences in the relationship between specific cognition function and mortality risk in younger populations. In our study, however, younger individuals with poor specific cognitive function and without dementia exhibited the same pattern. Although the accurate reason remains unknown, one possible explanation is that estrogens increase the formation of new excitatory synapses, influence development, enhance cholinergic function, and inhibit amyloid formation [30, 31], and these increased levels are associated with reduced mortality and cognitive decline among women [32, 33]. The protective effect of estrogen might also play an important role in the young premenopausal women of this study.

The biological mechanisms in the relationship between poor cognitive function and mortality remain unclear, although recent findings have provided plausible explanations. Poor cognitive function might be associated with mortality through causal pathways that are possibly modified by health behaviors [26], social factors [34], or a direct neuropathological mechanism [35]. In addition, people with poorer cognitive ability usually have fewer opportunities to attain a better socioeconomic position and may receive fewer health care resources, which is related to increased mortality [36]. Another possible explanation is that cognitive impairment is related to an increase in the risk of cardiovascular disease or stroke via lifestyle behaviors [37]. Lifestyle factors shown to be associated with an increased mortality risk include smoking, physical inactivity, abnormal blood pressure, blood glucose, total cholesterol levels, weight, and a poor diet [38]. In addition, Bostock and Steptoe noted that a lower ability to read, process, and understand basic health-related information was associated with higher mortality in older adults [39]. These results are consistent with the results of our study, showing that young male subjects with lower cognitive function had a higher risk of all-cause mortality compared to young male subjects with normal cognitive function. The particular cognitive processes of attention and short-term memory that were tested in our study ranged from general cognitive ability to efficient learning, problem solving, and reasoning. It is tempting to speculate that young adults with lower cognitive function may have lower health literacy, which is associated with an increased risk of mortality. In response to cognitive impairment, clinicians might perform specific interventions such as the use of plain language to disseminate health educational information to the elderly; however, less attention has been paid to interventions for younger patient populations.

Several limitations of this study are worth noting. First, only two types of cognitive function tests were included in our analysis, and different domains of cognitive processes may have an unequal effect on mortality. However, the cognitive domain of memory, which was tested in our study, has shown a leading role in the progression of dementia [40, 41], implying that memory may be the representative domain in cognitive function. Second, it is difficult to compare our results with previous studies due to the different measures of cognitive function used in different studies. Third, there is no accurate consensus regarding the delineation of individuals with varying degrees of cognitive function. Therefore, we compared the “inferior” group of cognitive performance scores to our baseline measurements, and the linear association between mortality and cognitive function should be tested in the future. Fourth, only single assessment of cognitive performance in people aged 20–59 was available; therefore, it is difficult to gauge whether lower scores of the tests are a reflection of decline or actually of peak level of cognition.

## 5. Conclusions

We found that poorer executive function and poorer short-term memory were associated with mortality in younger male adults. Significant prognostic information was provided using simple cognitive tests, and our results suggest that poorer cognitive function should also prompt a high clinical suspicion for the potential risk of mortality in younger males.

## Figures and Tables

**Table 1:** Demographic and baseline characteristics stratified by life status.

Variable	Survivors (*n* = 4,764)	Deceased (*n* = 380)	*P* value
Continuous variables, mean ± SD			
Age (year)	36.08 ± 10.74	44.34 ± 10.79	<0.001
Systolic blood pressure (mmHg)	117.54 ± 15.75	127.30 ± 20.28	<0.001
Diastolic blood pressure (mmHg)	72.52 ± 12.51	77.44 ± 14.13	<0.001
Serum folate (ng/mL)	5.62 ± 4.43	5.64 ± 4.13	0.939
Serum C-reactive protein (mg/dL)	0.42 ± 0.59	0.59 ± 0.82	<0.001
Serum glucose (mg/dL)	94.34 ± 27.53	108.45 ± 54.28	<0.001
Body mass index	27.15 ± 6.09	28.20 ± 7.15	<0.001

Categorical variables, *n* (%)			
Male	2,129 (44.69)	214 (56.32)	<0.001
Non-Hispanic White	1,686 (35.39)	132 (34.74)	0.01
Education (before high school)	953 (20.14)	104 (27.44)	0.001
Smoker	2,265 (47.54)	264 (69.47)	<0.001
History of chronic heart failure	34 (0.71)	23 (6.05)	<0.001
History of stroke	23 (0.48)	9 (2.37)	<0.001
History of chronic bronchitis	208 (4.37)	36 (9.47)	<0.001

Cognitive function test, mean ± SD			
Symbol digit substitution test	2.91 ± 1.13	3.65 ± 1.91	<0.001
Serial digit learning task: total score	5.95 ± 5.02	7.61 ± 5.36	<0.001

SD: standard deviation.

**Table 2:** Associations between cognitive function tests and all-cause mortality.

Cognitive function test	Male				Female			
	Model 1		Model 2		Model 1		Model 2	
	HR (95% CI)	*P* value	HR (95% CI)	*P* value	HR (95% CI)	*P* value	HR (95% CI)	*P* value
Symbol digit substitution test (SDST)	2.88 (2.06, 4.02)	<0.001^*∗*^	2.02 (1.36, 2.99)	0.001^*∗*^	2.64 (1.80, 3.86)	<0.001^*∗*^	1.36 (0.82, 2.25)	0.23
Serial digit learning task (SDLT)	1.75 (1.28, 2.40)	<0.001^*∗*^	1.47 (1.02, 2.12)	0.041^*∗*^	1.66 (1.15, 2.38)	0.006^*∗*^	1.23 (0.77, 1.95)	0.39

HR: hazard ratio; CI: confidence interval.

^*∗*^ *P* < 0.05.

Model 1: unadjusted.

Model 2: adjusted for age, race/ethnicity, education, blood pressure, serum folate, serum C-reactive protein, serum glucose, body mass index, smoking status, heart failure, stroke, and chronic bronchitis.

## Abbreviations

NAFLD: Nonalcoholic fatty liver disease
NHANES III: Third National Health and Nutrition Examination Survey
SDST: Symbol digit substitution test
DSST: Digit symbol substitution test
SDLT: Serial digit learning task
NES: Neurobehavioral Evaluation System
BMI: Body mass index
NDI: National Death Index.
